# A Review on Green Synthesis, Biomedical Applications, and Toxicity Studies of ZnO NPs

**DOI:** 10.1155/2018/3569758

**Published:** 2018-08-01

**Authors:** V. N. Kalpana, V. Devi Rajeswari

**Affiliations:** Department of Biomedical Sciences, School of Biosciences and Technology, VIT, Vellore, Tamil Nadu, India

## Abstract

The advance of reliable and eco-friendly strategies for the development of nanoparticles is a fundamental key to the discipline of nanotechnology. Nanoparticles have been continuously evaluated and have been used in many industrial applications for a decade. In particular, the role of zinc oxide nanoparticles (ZnO NPs) has received a great interest because of various properties such as UV filter properties and photochemical, antifungal, high catalyst, and antimicrobial activities. Because of the high rate of poisonous chemicals and the extreme surroundings used within the chemical and physical methods, the green techniques have been adopted using plants, fungi, bacteria, and algae for the synthesis of nanoparticles. Therefore, this paper considers various green synthesis methods to provide the evidence of ZnO NP role to several applications, and in addition, biomedical applications and toxic effect were reviewed. Therefore, the paper used various secondary sources to collect the relevant review articles. From the findings, the green route of synthesis is rather safe and eco-friendly when compared to physical and chemical means of synthesis. On the other hand, its biomedical applications in this sector are increased day by day in various processes including bioimaging, drug delivery, biosensors, and gene delivery. With respect to its toxicity properties, ZnO NPs can act as smart weapons against multiple drug-resistant microorganisms and as a talented substitute for antibiotics.

## 1. Introduction

In science and technology, one among the rapidly developing concepts in the latest years is nanotechnology, which has brought tremendous development. The nanomaterial which comprises distinctive physicochemical properties has the potential to develop new systems, structures, devices, and nanoplatforms with impending bids in extensive variety of disciplines [1, 2]. Nanomaterials are particles that are in nanoscale size, and they are very small particles with improved thermal conductivity, catalytic reactivity, nonlinear optical performance, and chemical stability due to their large surface area-to-volume ratio [3]. This quality has attracted many researchers to locate novel techniques for their synthesis. Though conventional techniques (physical and chemical methods) use less time to synthesize bulk amount of nanoparticles, they require toxic chemicals like protective agents to maintain stability, which leads to toxicity in the environment. Keeping this in mind, green technology by using plants is rising as an eco-friendly, nontoxic, and safe option, since plant extract-mediated biosynthesis of nanoparticles is economically advantageous and offers natural capping agents in the form of proteins [4]. To regulate chemical toxicity in the environment, biological synthesis of various metal oxide and metal nanoparticles through plant extraction is used, which is a marginal technique for regulating chemical synthesis, and it permits a distinct shape and size of nanoparticles with a meticulous synthesis [5].

For biomedical applications, improvement in biodegradable, functionalized, and biocompatible nanomaterials is being remained a tremendous vivacious area for research. Until now, among numerous other biomedical applications [6–9] well examined are paramagnetic nanoparticles [10, 11], quantum dots (QDs) [6, 12], nanoshells [13], and carbon nanotubes (CNTs) [14, 15]. An extensive variety of nanostructures could exhibit zinc oxide (ZnO), which has exclusive properties such as semiconducting, piezoelectric, and optical [16, 17]. Hence, nanomaterials based on ZnO are deliberated for extensive range of applications such as energy storage, nanosensors, cosmetic products, nano-optical devices, nanoelectronic devices, and so on [18–23]. Biodegradability and low toxicity are one among the most significant characteristics of ZnO nanomaterials. For adults, an indispensable trace element is Zn^2+^ and it is being elaborated into numerous features of metabolism. In acidic and strong basic conditions, ZnO NPs could be dissolved slowly. Solubilized ZnO nanoparticles have shown that the release of Zn^2+^ ions can exert stress on cells and have adverse impacts on different organisms [24]. The required properties of ZnO nanomaterials have attained increased concern towards biomedical applications [25]. The ZnO nanoparticle's toxic effect is due to their solubility. In the extracellular areas, ZnO nanoparticles are dissolved, which sequentially raise the level of intracellular [Zn^2+^]. The dissolution of ZnO nanoparticles in the medium and the mechanism of increased intracellular [Zn^2+^] level are still speculative [26].

Holistically, this review will summarize the current status of the use of ZnO nanomaterials for biomedical applications, their green synthesis nature, and its toxic effect.

Organic nanoparticles and inorganic nanoparticles are the two categories of nanoparticles based on their components. Organic nanoparticles include carbon nanoparticles (fullerenes), whereas inorganic nanoparticles include magnetic nanoparticles, noble metal nanoparticles (gold and silver), and semiconductor nanoparticles (such as zinc oxide and titanium dioxide) [27]. Nanoparticles can also be categorized based on their origin, dimensions, and structural content.

Based on the origin of nanomaterial, it is categorized into natural nanomaterial and artificial nanomaterial [28]. On considering the dimensions of nanomaterial, it is categorized as zero-dimensional (0D), one-dimensional (1D), two-dimensional (2D), and three-dimensional (3-D) nanomaterials. The zero-dimensional nanomaterials have nanodimensions at all three directions; one-dimensional nanomaterials have only one nanodimension which is exterior to the nanometer range; and two-dimensional nanomaterials have two nanodimensions exterior to the nanometer range, whereas three-dimensional nanomaterials have all the nanodimensions exterior to the nanometer range. These comprise bulk materials developed with individual blocks that are in the nanometer scale (1–100 nm) [29].

According to the structural configuration and morphology, nanomaterials have been divided into amalgamated materials and nanodispersions. Extremely branched macromolecules are dendrimers with the dimensions in the nanometer scale. In the metal-based materials, the chief component for these particles is metal, where the nanomaterials comprised nanosilver, nanogold, metal oxides like titanium dioxide, and finally closely packed semiconductors such as quantum dots. The morphology of carbon-based nanomaterials is tubes, hollow spheres, or ellipsoids. The carbon nanomaterials that are spherical and ellipsoidal are referred as fullerenes and cylindrical ones are called as nanotubes [28].

## 2. Nanoparticle Synthesis Methods

Bottom-up and top-down are the two approaches recommended for the biosynthesis of nanoparticles [30]. In case of a bottom-up approach, the most important reaction occurred is oxidation/reduction. The synthesis of nanoparticles is currently an important area of research, which seeks an eco-friendly approach and green materials for current scenario [31]. The major steps involved in the preparation of nanoparticles that have to be evaluated from the point of green chemistry are (i) the solvent medium used for the synthesis, (ii) environmentally benign reducing agent, and (iii) the nontoxic material for the stabilization of the nanoparticles. The majority of the chemical and physical methods mentioned so far largely depend on organic solvents. This is principally due to the hydrophobicity of the capping agents used [32]. Synthesis with bio-organisms is compatible with the principles of green chemistry: (i) eco-friendly approach, (ii) the reducing agent used, and (iii) the capping agent in the reaction. The synthesis of inorganic metal oxide nanoparticles using biological elements has received immense attention due to their unusual properties (optical, electronic, chemical, etc.) [33].

## 3. Zinc Oxide Nanoparticles

Metal oxides play a very significant role in the science of materials, such as the production of microelectronic circuits, sensors, piezoelectric devices, fuel cells, surface passivation coatings, and corrosion catalysts. Metal oxides have also been used as absorbers of the environmental pollutant. In nanotechnology, oxide nanoparticles can show signs of unique chemical properties due to their limited size and high density of edges. An n-type semiconducting metal oxide is ZnO. Over the past few years, more interest is drawn towards zinc oxide NP since it has wider varieties of applications particularly in the fields of biomedical systems, optics, and electronics [34–40]. Among all these types of metal oxides, ZnO NPs attract much attention because of their stimulating properties [41] (such as the high direct bandwidth of 3.3 eV at room temperature and high excitation energies of 60 meV) [42], optical property, high catalytic activity, anti-inflammatory, wound healing, and UV filtering properties [1, 43–48]. Several authors have reported ZnO biosensors for cholesterol, enzyme biochemistry, and other biosensing applications [49, 50].

Zinc oxide as a nonhygroscopic and nontoxic, inorganic, polar, crystalline material is a very cheap, safe, and readily available, which has aroused great interest in various organic transformations, sensors, transparent conductors, and surface acoustic wave devices [51–53]. The ZnO NP is an exclusive material that has semiconducting, piezoelectric, and pyroelectric properties and has versatile applications in transparent electronics, UV light emitters, chemical sensors, spin electronics, personal care products, catalyst, coating, and paints [54, 55]. Due to these unique properties, ZnO NPs find applications in antireflection coatings, transparent electrodes in solar cells, UV light emitters, diode lasers, varistors, piezoelectric devices, spin electronics, surface acoustic wave propagators [52], as an antibacterial agent [56], as photonic material [57], and for gas sensing [58]. The biomolecules in the plant extract act as efficient capping agents, thus playing a major role in the NP synthesis. The capping agents seem to stabilize NPs through different mechanisms that include electrostatic stabilization, steric stabilization, stabilization by hydration forces, and stabilization using van der Waals forces. The stabilization of NPs is significant for its functions and its applications [59]. The utility of ZnO NPs in the field of food preservation and packaging industry when applied to biodegradable polymeric metrics and ZnO NPs has improved the quality of food and packaging mainly through three mechanisms, namely, the release of antimicrobial ions, destructing the integrity of the cells of bacteria, and formation of ROS due to light radiation [60]. Elmer and White reported the pesticidal properties of the ZnO by spraying synthesized ZnO on tomato and eggplant and it was noted that ZnO reduced disease estimate of 28% when compared to the control [61]. In vitro approaches use plant extracts to reduce zinc salt (zinc nitrate, sulfate, chloride, and many others) and endow with control over the size and shape of the nanoparticles. Fundamentally, the primary and secondary metabolites are present in plants, for example, saponins, tannins, starches, polypeptides, terpenoids, flavonoids, and phenolics, which act as reducing and capping agents. Mild solvents such as water, ethanol, and methanol are used for the extraction of the plant metabolites, which are allowed to react with the zinc salt solution in different conditions to achieve greatest yield [62–65].

## 4. Green Synthesis of ZnO NPs

Owing to the growing popularity of green methods, several methods have been implemented to produce ZnO NPs using different sources such as bacteria, fungus, algae, plants, and others. A list of tables was prepared to summarize the research carried out in this field (Table 1).

### 4.1. Plant-Mediated Biosynthesis of ZnO NPs

**Figure 1:**
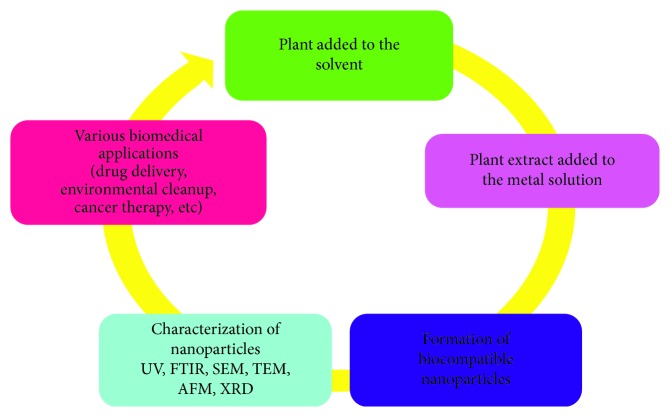
Plant-mediated biosynthesis of ZnO NPs.

The synthesis of biological nanoparticles represents an alternative for the physical and chemical methods of nanoparticle formation. The majority of researchers focused on the green synthesis of nanoparticles for the formation of metal and oxide nanoparticles (Figure 1). The use of plants for the synthesis of nanoparticles is a rapid, low-cost, eco-friendly option and is safe for human use [31].

*Vitex negundo* plant extract was used to produce ZnO NPs with zinc nitrate hexahydrate as a precursor. The biosynthesized ZnO NPs showed antimicrobial activities against *E. coli* and *S. aureus* bacteria [77]. Dobrucka and Dugaszewska [78] used *Trifolium pratense* to synthesize ZnO NPs. The synthesized ZnO NPs were found to be of hexagonal shape and the sizes were found to be of 60–70 nm. Kalpana et al. [79] synthesized ZnO NPs using *Lagenaria siceraria* pulp extract. In addition, the author evaluated the biosynthesized ZnO nanoparticles for antidandruff, antimicrobial, and antiarthritic efficacy.

Dhanemozhi et al. [80] successfully synthesized the ZnO NPs from green tea leaf extract to evaluate their capacitance behavior for supercapacitor applications. ZnO NPs are known to be multifunctional inorganic nanoparticles with their major application in the treatment of urinary tract infection. Santhoshkumar et al. [81] synthesized the ZnO NPs using *Passiflora caerulea* leaf extract and tested against the pathogenic culture isolated from the urine of the patient suffering from urinary tract infection. The results showed that the synthesized ZnO NPs act as an antibacterial agent against urinary tract infection. Nava et al. [82] dealt with low-cost, nontoxic green synthesis of ZnO NPs prepared using *Camellia sinensis* extract. The efficiency of ZnO NPs as a photocatalyst for the degradation of various organic dyes such as methylene blue and methyl orange and their antioxidant activity by the DPPH assay has been studied by Siripireddy and Mandal [83] using *Eucalyptus globulus*. The synthesis of monophase crystalline ZnO nanoparticles with a size range of about 15.8 nm by the green novel and environmentally friendly pathway using the extract of *A. betulina* as an effective oxidizing/reducing agent has been demonstrated for the first time by Thema et al. [84]. Stable and spherical ZnO NPs were produced by using zinc nitrate and *Aloe vera* leaf extract. The various properties of ZnO NPs were characterized by the use of the UV-Vis spectrophotometer, FTIR, photoluminescence, XRD, SEM, and TEM analysis [43].

### 4.2. Microbe-Mediated Biosynthesis of ZnO NPs

Synthetic pathways of nanoparticles by microbes may involve combinations of basic cell biochemistry, the transport of ionic metals both in and out of cells, mechanism of resistance of microbes to toxic metals and activated metal-binding sites, ion accumulation metallic intracellular, and nucleation of metal oxides [85]. ZnO NPs were rapidly synthesized from a *Rhodococcus pyridinivorans* NT2 which were found to be moderately stable and roughly spherical with an average diameter of the particle of 100–120 nm [86]. *Serratia ureilytica* (HM475278)-mediated ZnO NPs have been reported by Dhandapani et al. [87]. ZnO NPs have aroused interest because of their many applications in the food industry. Selvarajan and Mohanasrinivasan [88] described an innovative method for the biosynthesis of ZnO NPs using a probiotic bacterium *Lactobacillus plantarum* VITES07. Kundu et al. [86] synthesized ZnO NPs from the zinc sulfate solution using an actinobacteria *Rhodococcus pyridinivorans* NT2. The synthesized ZnO NPs were explored for multifunctional textile finishing and for in vitro anticancer drug delivery in HT-29 colon carcinoma cell line. The studies of Shamsuzzaman et al. [89] have described the simple and green route for the biosynthesis of ZnO nanoparticles using *Candida albicans* as a capping and reducing agent. The author also used the synthesized ZnO NPs as a catalyst for the rapid and efficient synthesis of steroidal pyrazoline. Hussein et al. [85] reported *Bacillus cereus* as a biotemplate agent for the formation of ZnO NPs with raspberry and plate-like structures through a simple thermal decomposition of zinc acetate maintaining the original pH of the reaction mixtures. Baskar et al. [90] produced ZnO NPs using *Aspergillus terreus* filtrate synthesized extracellularly which were spherical with a range of 54.8 to 82.6 nm.

The nanoparticle's extracellular synthesis from the fungus is extremely beneficial due to economic viability, convenient downstream processing, and large-scale production [91]. Fungal strains are preferred over bacterial due to their enhanced metal bioaccumulation property and tolerance property [92]. From mycelia of *Aspergillus fumigatus*, the ZnO nanoparticles are synthesized [93]. By utilizing *Candida albicans*, the nanoparticles were synthesized with similar size ranging from 15 to 25 nm established through XRD, TEM, and SEM analysis [89].

## 5. Characterization of ZnO NPs

The synthesized nanoparticles are characterized by utilizing numerous techniques: FTIR (Fourier transform infrared spectroscopy), EDAX (energy dispersion analysis of X-ray), AFM (atomic force microscopy), XPS (X-ray photoelectron microscopy), ATR (attenuated total reflection), UV-DRS (UV-visible diffuse reflectance spectroscopy), XRD (X-ray diffractometer), TEM (transmission electron microscopy), TG-DTA (thermogravimetric-differential thermal analysis), DLS (dynamic light scattering), FE-SEM (field emission scanning electron microscopy), PL (photoluminescence analysis), Raman spectroscopy, and SEM (scanning electron microscopy) [94–96]. Plants are being examined extensively particularly that belong to Lamiaceae family such as *Vitex negundo* [97], *Plectranthus amboinicus* [98], and *Anisochilus carnosus* [37] which had the formation of NP with different shapes such as hexagonal, rod-shaped with agglomerates, quasispherical, and spherical, and further various sizes are also seen. From the outcome, it is clearly identified that the size of synthesized NPs is decreased on increasing the concentration of a plant extract [70, 77]. From the results, the size range is being observed and compared using various techniques such as TEM, XRD, and FE-SEM which had a closer range of values [77, 98], whereas SEM and EDAX had a similar result diverse from XRD results. Through Debye–Scherrer equation, synthesis of NPs from both *Vitex negundo* flower and leaf had a similar size of 38.17 nm, which was confirmed through XRD analysis [77]. For the synthesizing ZnO NPs, leaves of *Azadirachta indica* from Meliaceae family are generally being used [99, 100]. A similar size range of NPs was identified in every experiment, which was confirmed through the analysis of TEM and XRD with nanobuds, hexagonal disc shape, and spherical shape. From the studies, it is revealed that the formation of NPs is through the involvement of amine, carboxylic acid, carbonate moieties, alcohol, alkane, and amide, which was further confirmed by FTIR studies. *Aloe vera's* leaf peel and fresh leaf extract belong to Liliaceae family [101, 102]. Agglomerate formation was seen in the NP synthesis, which was extracted from *Moringa oleifera*, *Calotropis gigantea*, *Plectranthus amboinicus*, *Agathosma betulina*, *Nephelium lappaceum*, and *Pongamia pinnata*. To affirm the synthesis of NPs, UV-Vis spectrophotometry is employed, and the crystal NPs are obtained through centrifugation of mixture and drying the pellet in a hot air oven [95].

## 6. Biomedical Applications of ZnO NPs

### 6.1. Drug Delivery

The benefits of using ZnO NPs for the drug delivery were derived from their two main basic properties. First of all, due to their smaller size, nanoparticles can penetrate through smaller capillaries and are absorbed by the cells, allowing an efficient accumulation of drugs at the target sites. Second, the use of biodegradable materials for the preparation of nanoparticles allows the prolonged discharge of drugs within the site targeted over a period of days or even weeks [103]. The role of synthesized ZnO NPs in drug release by using the drug metronidazole benzoate was studied [68] by observing its diffusion through egg membrane. Results revealed that the presence of ZnO NPs with the drug has much effect on the biological membrane.

### 6.2. Bioimaging of ZnO Particles

In preclinical research, fluorescence imaging is extensively utilized as it is convenient and inexpensive [104–107]. As ZnO nanomaterials have essential excitonic blue and near-UV emission, which has green luminescence associated with O_2_ vacancies [108, 109], and for cellular imaging, there are numerous reports existing in previous studies on the utilization of ZnO nanomaterials. For cancer cell imaging, transferrin-conjugated green fluorescent ZnO NPs were utilized with least cytotoxicity [110]. ZnO nanomaterial's optical properties could be altered by adulterating with suitable elements [111]. According to a research, ZnO NPs were adulterated with various cations such as Co, Cu, or Ni, and in aqueous colloidal solutions, it was stabilized, which was employed in different cells for cellular imaging studies [112]. These tiny ZnO nanoparticles have a capability to penetrate it into the cell nucleus.

For biocompatibility and optical properties, heterostructural ZnO/Au nanocomposites, where Au NCs develop either along the nanorod surfaces or at the tip of ZnO nanorods, are synthesized and investigated [113]. For imaging of cancer cells in vitro, antiepidermal growth factor receptor antibody-conjugated ZnO nanorods were utilized in the latest research [114]. For optical imaging, QDs are extensively deliberated nanoparticles because of their numerous appealing optical properties [115–117]. It was identified that ZnO QDs are placed in the cytoplasm while applying for in vitro cell imaging, exhibiting stable luminescence under UV light in the absence of essential cytotoxicity. Same QDs were analyzed in a previous research which was trialed through mice after injecting intravenous and intradermal injections [118].

Every imaging technique has their own benefits and drawbacks [119]. Through multiple imaging modalities, nanomaterials could be functionalized to be detectable, which produce synergistic advantages. Nanomaterials are more appropriate for multimodality imaging while associated with small molecules since larger surface area provides higher sites for functionalization and also helps to engineer them for multimodal detection. In one particular research, Gd-doped ZnO QDs (with sizes of less than 6 nm) were emerged for both magnetic resonance imaging (MRI) and optical imaging [120]. Another study by Singh reported Fe_3_O_4_-ZnO core-shell magnetic QDs for potential cancer imaging and therapy.

There are better clinical relevance for single-photon emission computed tomography (SPECT) [122–126] and radionuclide-based imaging techniques, that is, PET [127–132], which are extensively utilized in the clinic than in optical imaging. PET and SPECT techniques are not only highly sensitive and quantitative but also have no tissue penetration limitation [133–136].

### 6.3. Drug Delivery with ZnO Nanomaterials

ZnO nanomaterials are versatile nanoplatforms not only in bioimaging but also in a drug delivery application because of their versatile surface chemistry, large surface area, and phototoxic effect, along with others. Researches in vitro have identified that ZnO nanoparticles could be highly toxic either for cancer cells [126] or for bacteria and leukemic T cells [137].

Intrinsic blue fluorescence of ZnO QDs was smeared with folate-conjugated chitosan through electrostatic interaction, and by doxorubicin, it can be loaded at ∼75% efficiency (extensively utilized chemotherapy drug is DOX) [138]. It was recommended that through hydrogen bonding and/or through collaborations with the ZnO QD surface, DOX was entrapped. But the aqueous stability of the ZnO QDs enriched the exterior chitosan layer because of the hydrophilicity and the charges. Conversely, at the normal physiological pH value of 7.4, DOX was released rapidly which requires to be improved for investigations in in vivo or in vitro researches.

In dendritic cell- (DC-) established cancer immunotherapy, one of the main complications is the improvement of delivery system which could provide the targeted antigens into DCs efficiently [139]. Due to extensive surface area, nanomaterials are challenging aspects for this application.

To deliver carcinoembryonic antigen into DCs, Fe_3_O_4_-ZnO core-shell nanoparticles were produced with an average diameter of 16 nm which have the capability to help as imaging contrast agents [140].

### 6.4. Gene Delivery with ZnO Nanomaterials

Over the last few years, gene therapy has involved substantial attention over cancer treatment [141]. Developing a safe gene vectors which could safeguard DNA from degradation as well as through high efficiency enabling cellular uptake of DNA is one of the foremost challenging aspects. For examining gene therapy application and gene delivery, extensive varieties of nanomaterials are utilized, which even comprise ZnO nanomaterial that had a positive outcome in numerous studies.

By a sequence of investigations, ZnO nanostructures, which are also like a three-dimensional tetrapod, were examined as gene vectors for delivering pEGFPN1 DNA (comprising the gene for green fluorescent protein) to A375 human melanoma cells [142, 143]. Through electrostatic interactions, the pDNA (plasmid DNA) was attached to ZnO nanostructures, and for gene delivery within the cells, the three needle-shaped legs preferred the internalization of the tips. It was observed that there was an absence for significant cytotoxicity which was reportedly attributed for the three-dimensional geometry.

For an efficient gene delivery, surface coating of nanomaterial acts as a significant role. According to an investigation, ZnO QDs were layered using positively charged poly(2-(dimethylamino)ethyl methacrylate) (PDMAEMA) polymers which are utilized for condensing pDNA for gene delivery [144]. The polymer-coated ZnO QDs presented fluorescence emission at 570 nm with a considerable amount of less than 20% which is capable of condensing large pDNA just like luciferase reporter gene. It was stated that COS-7 cells can be transfected proficiently with pDNA transmitting ZnO QDs with lower cytotoxicity. The ZnO QDs had a significantly decreased cytotoxicity in association with the application of PDMAEMA as the gene vector. The decrease in cytotoxicity was due to the existence of negatively charged polymethacrylate in the QDs which stabilized the positive charges.

### 6.5. Biosensors based on ZnO Nanomaterials

Biosensors are extensively utilized in food industry, environmental monitoring, and healthcare and in chemical or biological analysis. Examples for biosensors are electrochemical, photometric, piezoelectric, and calorimetric among others when categorized based on the detection principles [145].

Nanomaterials, either as uncombined or as combination with biologically active substances, are attaining ever-increasing awareness because of their capability to deliver a suitable platform for developing high-performance biosensors, which is due to their distinctive features [23]. For instance, the higher surface area of nanomaterials could be utilized for immobilizing numerous biomolecules such as antibodies, enzymes, and other proteins. Moreover, they could be permitted for a direct electron transfer among the electrodes and the active sites of the biomolecules.

ZnO nanomaterials also provide numerous desirable traits apart from semiconducting properties such as biosensing, strong adsorption capability, high isoelectric point, and high catalytic efficiency (IEP; ∼9.5) which are appropriate for adsorption of certain proteins such as antibodies and enzymes with less IEPs by electrostatic interaction [146]. Moreover, the favorable conditions of nanomaterials to be used in biosensors are lower toxicity, higher electron transfer capability, higher surface area, and better biocompatibility or stability [147]. The most widely stated ZnO-based biosensors are recognized for numerous small-molecule analytes such as cholesterol, phenol, urea, glucose, H_2_O_2_, and many more other things. Additionally, there are numerous biosensors for sensing other molecules and certain physical or chemical properties like pH [148, 149].

## 7. Toxicity Studies of ZnO NPs

Due to the increasing use of nanoparticles and their release in the environment, it is necessary to determine the toxicity of nanoparticles. Vicario-Pares et al. [150] conducted a toxicity study of three metal oxide nanoparticles, namely, CuO NPs (copper oxide nanoparticles), ZnO NPs, and TiO_2_ NPs against zebra fish embryo. ZnO NPs were found to be less toxic than the ionic form of zinc, which exerts the highest toxicity. Studies of Zhu et al. [151] showed that ZnO NP toxicity is dose dependent. Similarly, Jeyabharathi et al. [152] evaluated the toxicity study of green synthesized ZnO NPs towards zebra fish embryos. The author synthesized ZnO NPs from *Amaranthus caudatus* leaf extract. Further, ZnO NPs were found to exhibit a higher antibacterial activity against *Staphylococcus epidermidis* and *Enterobacter aerogenes*. Results of the toxicity study show that ZnO NPs at a concentration of 10 mg/ml did not show any significant effect on survival and malformation in the zebra fish embryo. In a 90-day toxicity study, 100 nm ZnO NPs with different surface charges (negatively charged, ZnOAE100 [−] and positively charged ZnOAE100 [+]) were administered to Sprague Dawley rats to determine the toxic level and to identify target organs. Significant toxic effects were observed in both sexes at a concentration greater than 125 mg/kg. Also, there was an absence of adverse effect level at a concentration of about 31.25 mg/kg for both sexes [153].

## 8. Conclusions

Overall, from the study reviews, it is considered that green ZnO NP synthesis is much safer and environmentally friendly than the physical and chemical methods. ZnO NPs are one of the most important and versatile materials, due to their diverse properties, functionalities, various benefits, and applications to humans. The green sources act as a stabilizing and reducing agent for the synthesis of nanoparticles of controlled size and shape. Holistically, the ZnO NP application to crops increases the growth and yield in agriculture. As demand for food is increasing day by day, the yield of a staple crop is low. Thus, it is necessary to commercialize metal oxide nanoparticles for sustainable agriculture. On the other hand, its biomedical applications in this sector are increased day by day in various processes including bioimaging, drug delivery, biosensors, and gene delivery. With respect to its toxicity properties, ZnO NPs can act as smart weapons against multiple drug-resistant microorganisms and as a talented substitute for antibiotics. It is anticipated that this review could further streamline the research on innovative methodological and clinical correlations in this area. In the meantime, solutions to health problems will be suggested by referring to this complex through scientific and research reports.

## Figures and Tables

**Table 1:** Green synthesis of ZnO NPs using various sources.

Type of green method	Applied material/organism	Particle size (nm)	Morphology of NPs	Activity carried out	References
Plant-mediated synthesis	*Limonia acidissima* (leaf)	12–53	Spherical	Antibacterial activity against *Mycobacterium tuberculosis*	[39]
	*Euphorbia Jatropha* (stem)	15	Hexagonal	Used as semiconductors	[66]
	*Ceropegia candelabrum* (leaf)	12–35	Hexagonal	Antibacterial potential against *Staphylococcus aureus*, *Bacillus subtilis*, *Escherichia coli*, *Salmonella typhi*	[67]
	*Celosia argentea* (leaves)	25	Spherical	Antibacterial potential against *Escherichia coli*, *Salmonella*, *Acetobacter*; drug delivery	[68]
	*Couroupita guianensis* (leaves)	57	Hexagonal unit cell	*Bacillus cereus*, *Klebsiella pneumonia*, *Escherichia coli*, *Mycobacterium luteus*, *V. cholerae*	[69]
	*Allium cepa* (bulb), *Allium sativum* (bulb), *Petroselinum crispum* (leaves)	70	Hexagonal wurtzite	Photodegradation of methylene blue	[48]
	*Phyllanthus niruri* (leaves)	25.61	Quasispherical	Catalytic activity	[70]
	Parthenium hysterophorus (leaves)	27–84	Spherical and hexagonal	*Aspergillus flavus*, *Aspergillus niger*, *Aspergillus fumigatus*, *Fusarium culmorum*, *Fusarium oxysporum*	[71]
	*Solanum nigrum* (leaves)	29	Quasispherical	*Staphylococcus aureus Salmonella paratyphi*, *Vibrio cholerae*	[72]
	*Anisochilus carnosus* (leaves)	20–40	Hexagonal wurtzite	*S. paratyphi*, *V. cholerae*, *S. aureus*, and *E. coli*	[37]
	*Jacaranda mimosifolia* (flower)	2–4	Hexagonal wurtzite	*Escherichia coli*, *Enterococcus faecium*	[73]

Seaweed-mediated synthesis	*Caulerpa peltata*, *Hypnea valencia*, *Sargassum myriocystum*	36	Rectangle, triangle, radial, and spherical	Antibacterial activity against *Staphylococcus aureus*, *Streptococcus mutans*, *Vibrio cholerae*, *Neisseria gonorrhoeae*, *Klebsiella pneumonia*, and antifungal activity against *Aspergillus niger* and *Candia* sp.	[74]
	*Ulva lactuca*	10–50	Hexagonal, rods, and rectangles	Photocatalytic, antibacterial, antibiofilm, and larvicidal activity	[75]

Microbe-mediated synthesis	*Aspergillus fumigatus*	60–80	Spherical	Antibacterial activity	[76]
	*Aeromonas hydrophila*	57	Spherical and oval	Antimicrobial activity	[41]
